# Supplementary material for: Metabolic modeling elucidates phenformin and atpenin A5 as broad-spectrum antiviral drugs against RNA viruses
Source: Commun Biol. 2025 May 23;8:791. doi: 10.1038/s42003-025-08148-y (PMC12102274; doi:10.1038/s42003-025-08148-y)
Supplement: Supplementary file 1 — Supplementary Information [file 42003_2025_8148_MOESM1_ESM.pdf]

Supplementary Information

Supplementary Figures

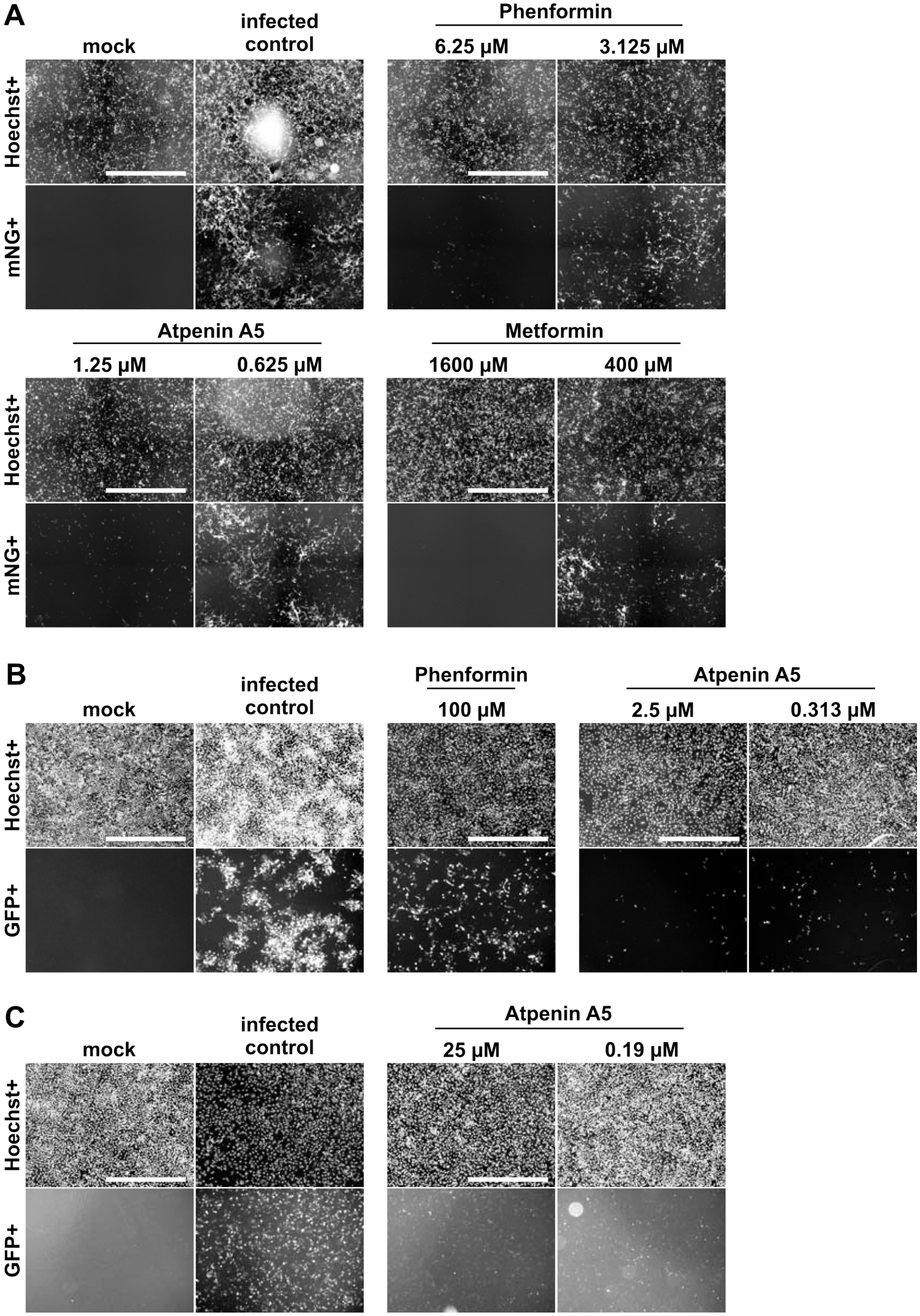

Supplementary Figure 1 | Primary data of infection experiments showing inhibition of SARS-CoV-2, RSV, and IAV by atpenin A5, phenformin, and metformin. Images show **A** Calu-3 cells infected with icSARS-CoV-2-mNG **B** A549 cells infected with the respiratory syncytial virus and **C** A549 cells infected with influenza A virus treated with the indicated compounds and concentrations. Scale bar is 1μm.

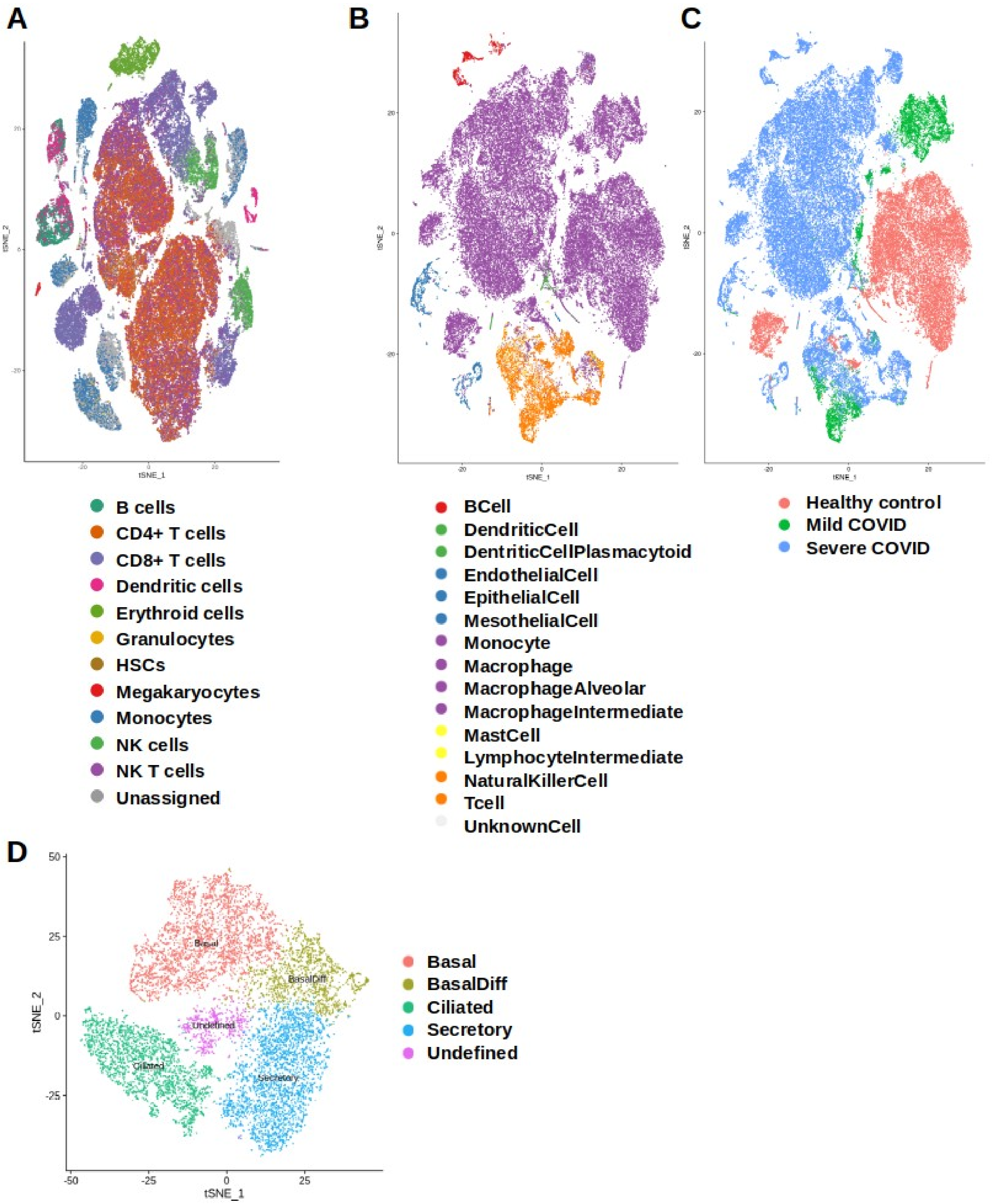

Supplementary Figure 2 | tSNE plots of cell types for the individual single-cell expression datasets of viral infection. Panels show **A** CHETAH-derived cell type annotation of the dengue dataset, based on a PBMC reference, and **B** CHETAH-derived cell type annotation of the BALF1 dataset, based on a single cell lung atlas. Panel **C** shows the same tSNE-plot as **B** of the BALF1 dataset but color coded for donors' health or COVID status. **D** Manual cell type annotation of the influenza H1N1 dataset.
