# Supplementary material for: Metabolic modeling elucidates phenformin and atpenin A5 as broad-spectrum antiviral drugs against RNA viruses
Source: Commun Biol. 2025 May 23;8:791. doi: 10.1038/s42003-025-08148-y (PMC12102274; doi:10.1038/s42003-025-08148-y)
Supplement: Supplementary file 2 — Description of Additional Supplementary Files [file 42003_2025_8148_MOESM2_ESM.docx]

**Description of Additional Supplementary Files**

File name: Supplementary Data 1

Description: Metabolic map of predicted shared tier-1 targets (blue) and broad-spectrum antiviral targets (red) across human metabolism. The map was created following the SBGN standard 116 using Recon2Map 117 based on the MINERVA platform 118.

File name: Supplementary Data 2

Description: Viral replication rates predicted tier-1/tier-2 targets and metadata for all reconstructed context-specific models.

File name: Supplementary data 3
Description: Figure source data

File name: Supplementary data 4
Description: Differentially active pathways in SARS-CoV-2 infected individuals

File name: Supplementary data 5
Description: Virus-host-interactions

File name: Supplementary data 6
Description: High-confidence tier-1 and tier-2 targets (Fig. 3)

File name: Supplementary data 7
Description: Frequency of tier-1 targets

File name: Supplementary data 8
Description: Broad spectrum antiviral targets

File name: Supplementary data 9
Description: Viral protein copy numbers

File name: Supplementary data 10
Description: Viral replication reactions

File name: Supplementary data 11
Description: Blood Serum constraints

File name: Supplementary data 12
Description: Dengue dataset cell type annotation
