# Supplementary figures and images for: Metabolic modeling elucidates phenformin and atpenin A5 as broad-spectrum antiviral drugs against RNA viruses
Source: Commun Biol. 2025 May 23;8:791. doi: 10.1038/s42003-025-08148-y (PMC12102274; doi:10.1038/s42003-025-08148-y)

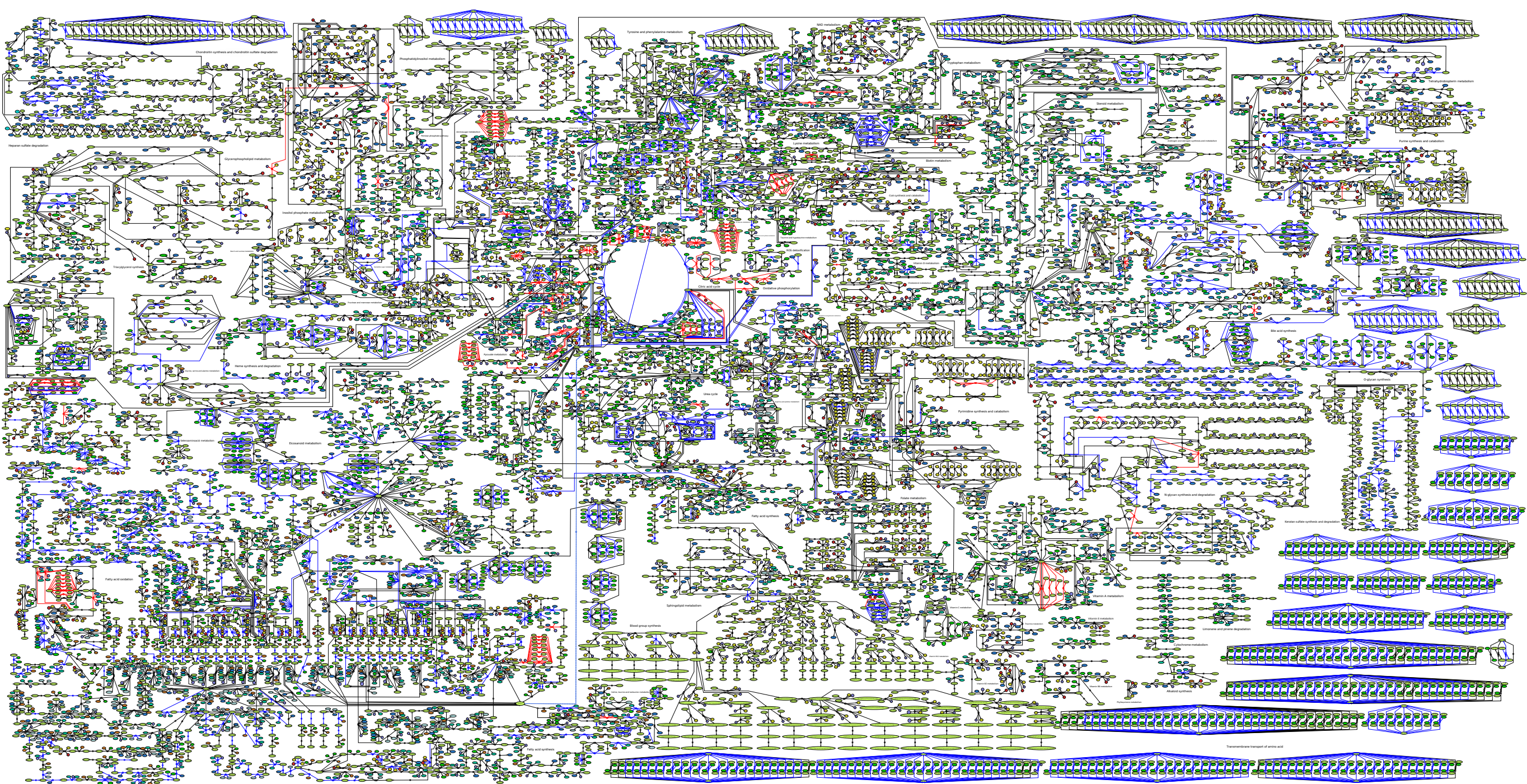

Supplement: Supplementary file 3 — Supplementary Data 1 [file 42003_2025_8148_MOESM3_ESM.zip › Supplementary_Data_1.pdf]
