# Supplementary material for: Metabolic modeling elucidates phenformin and atpenin A5 as broad-spectrum antiviral drugs against RNA viruses
Source: Commun Biol. 2025 May 23;8:791. doi: 10.1038/s42003-025-08148-y (PMC12102274; doi:10.1038/s42003-025-08148-y)
Supplement: Supplementary file 7 — Reporting Summary [file 42003_2025_8148_MOESM7_ESM.pdf]

Corresponding author(s): Andreas Dräger, Michael Schindler, Christoph Kaleta

Last updated by author(s): Jul 4, 2024

Reporting Summary

Nature Portfolio wishes to improve the reproducibility of the work that we publish. This form provides structure for consistency and transparency in reporting. For further information on Nature Portfolio policies, see our Editorial Policies and the Editorial Policy Checklist.

Statistics

For all statistical analyses, confirm that the following items are present in the figure legend, table legend, main text, or Methods section.

n/a	Confirmed	
☐	☒	The exact sample size (*n*) for each experimental group/condition, given as a discrete number and unit of measurement
☐	☒	A statement on whether measurements were taken from distinct samples or whether the same sample was measured repeatedly
☐	☒	The statistical test(s) used AND whether they are one- or two-sided<br>*Only common tests should be described solely by name; describe more complex techniques in the Methods section.*
☐	☒	A description of all covariates tested
☐	☒	A description of any assumptions or corrections, such as tests of normality and adjustment for multiple comparisons
☐	☒	A full description of the statistical parameters including central tendency (e.g. means) or other basic estimates (e.g. regression coefficient) AND variation (e.g. standard deviation) or associated estimates of uncertainty (e.g. confidence intervals)
☒	☐	For null hypothesis testing, the test statistic (e.g. *F*, *t*, *r*) with confidence intervals, effect sizes, degrees of freedom and *P* value noted<br>*Give P values as exact values whenever suitable.*
☒	☐	For Bayesian analysis, information on the choice of priors and Markov chain Monte Carlo settings
☒	☐	For hierarchical and complex designs, identification of the appropriate level for tests and full reporting of outcomes
☐	☒	Estimates of effect sizes (e.g. Cohen's *d*, Pearson's *r*), indicating how they were calculated

Our web collection on statistics for biologists contains articles on many of the points above.

Software and code

Policy information about availability of computer code

Data collection	Data was obtained from public repositories including Gene Expression Omnibus and original publications as detailed in Table 2.
Data analysis	Versions of Software used for analysis are provided in the methods section of the manuscript.

For manuscripts utilizing custom algorithms or software that are central to the research but not yet described in published literature, software must be made available to editors and reviewers. We strongly encourage code deposition in a community repository (e.g. GitHub). See the Nature Portfolio guidelines for submitting code & software for further information.

Data

Policy information about availability of data

All manuscripts must include a data availability statement. This statement should provide the following information, where applicable:

- Accession codes, unique identifiers, or web links for publicly available datasets
- A description of any restrictions on data availability
- For clinical datasets or third party data, please ensure that the statement adheres to our policy

All data produced in the context of this work is available in the Supplementary Tables and Data. Scripts are available via GitHub in the repository https://github.com/draeger-lab/R-DRUGS. The human metabolic models with all constraints for the blood medium and the viral replication reactions have been uploaded to the BioModels database: Dengue, https://www.ebi.ac.uk/biomodels/MODEL2308280001; Influenza, https://www.ebi.ac.uk/biomodels/MODEL2308280002; SARS-CoV-2, https://www.ebi.ac.uk/biomodels/MODEL2308280003.

Research involving human participants, their data, or biological material

Policy information about studies with human participants or human data. See also policy information about sex, gender (identity/presentation), and sexual orientation and race, ethnicity and racism.

Reporting on sex and gender	NA
Reporting on race, ethnicity, or other socially relevant groupings	NA
Population characteristics	NA
Recruitment	NA
Ethics oversight	NA

Note that full information on the approval of the study protocol must also be provided in the manuscript.

Field-specific reporting

Please select the one below that is the best fit for your research. If you are not sure, read the appropriate sections before making your selection.

☒ Life sciences ☐ Behavioural & social sciences ☐ Ecological, evolutionary & environmental sciences

For a reference copy of the document with all sections, see nature.com/documents/nr-reporting-summary-flat.pdf

Life sciences study design

All studies must disclose on these points even when the disclosure is negative.

Sample size	No sample size calculation was performed. Sample sizes were chosen according to the requirements of the given experiment including negative and positive controls.
Data exclusions	No data was excluded from the analyses.
Replication	For all experiments, at least three independent biological replicates were performed. Furthermore, we regularly include technical duplicates or triplicates to control for intraexperimental variations.
Randomization	For animal studies, subjects were randomized by weight on reception, in order to have the closest possible means between groups.
Blinding	For animal studies, the investigators were blinded for group allocation (treatment vs vehicle) during treatment administration, sampling, data collection, and viral load determination.

Reporting for specific materials, systems and methods

We require information from authors about some types of materials, experimental systems and methods used in many studies. Here, indicate whether each material, system or method listed is relevant to your study. If you are not sure if a list item applies to your research, read the appropriate section before selecting a response.

Materials & experimental systems

n/a	Involved in the study	
☒	☐	Antibodies
☐	☒	Eukaryotic cell lines
☒	☐	Palaeontology and archaeology
☐	☒	Animals and other organisms
☒	☐	Clinical data
☒	☐	Dual use research of concern
☒	☐	Plants

Methods

n/a	Involved in the study	
☒	☐	ChIP-seq
☒	☐	Flow cytometry
☒	☐	MRI-based neuroimaging

Eukaryotic cell lines

Policy information about cell lines and Sex and Gender in Research

Cell line source(s)	All cell lines were bought from ATCC or the German Collection of Microorganisms and Cell Cultures GmbH (DSMZ)
Authentication	Cell lines obtained from ATCC or DSMZ are authenticated by the respective insitute.
Mycoplasma contamination	All cell lines are regularly (bi-monthly) tested for mycoplasma and are tested negative.
Commonly misidentified lines (See ICLAC register)	NA

Animals and other research organisms

Policy information about studies involving animals; ARRIVE guidelines recommended for reporting animal research, and Sex and Gender in Research

Laboratory animals	Golden Syrian Hamster from Janvier Labs (France)
Wild animals	NA
Reporting on sex	Sex was originally considered in the animal study design, given the potential differences in COVID19 pathology. However, since this is a first in vivo POC study with only 16 animals and sex has not been reported as a significant variable in terms of treatment-dependent viral clearance during the acute infection phase (the main measured outcome), we only used animals of the same sex (female) to avoid unnecessary sources of heterogeneity. Future studies on treatment optimization (dosage, posology, long term effects, etc) warrant evaluation in both male and female animals.
Field-collected samples	NA
Ethics oversight	The animal experimental protocol was authorized by the Institutional Ethics Committee of VetAgro Sup (CEEA 18, project number 2066) and the French Ministry (APAFIS#27797-2020100516408472).

Note that full information on the approval of the study protocol must also be provided in the manuscript.
